# Atezolizumab-Induced Lambert-Eaton Myasthenic Syndrome in a Patient With Small-Cell Lung Cancer

**DOI:** 10.7759/cureus.33557

**Published:** 2023-01-09

**Authors:** Masami Yamazoe, Taku Hatakeyama, Kento Furukawa, Koji Kato, Kazuhiro Horiuchi

**Affiliations:** 1 Pulmonology, Hakodate Municipal Hospital, Hakodate, JPN; 2 Pulmonology, Sapporo Medical University School of Medicine, Sapporo, JPN; 3 Neurology, Hakodate Municipal Hospital, Hakodate, JPN

**Keywords:** immune-related adverse event, immune checkpoint inhibitor, atezolizumab, small-cell lung cancer, lambert-eaton myasthenic syndrome

## Abstract

The case of a 70-year-old man who developed Lambert-Eaton myasthenic syndrome (LEMS) while receiving *atezolizumab* treatment for extensive-stage small-cell lung cancer (SCLC) is presented. He started receiving maintenance immunotherapy with atezolizumab following four cycles of combination therapy with *atezolizumab*, carboplatin, and etoposide. After five cycles of maintenance *atezolizumab* therapy, he complained of muscle weakness in the lower limbs and fatigue. Electromyographic findings and positive results for anti-P/Q-type voltage-gated calcium channel antibody made a diagnosis of LEMS. Based on the onset time of LEMS and the state of his underlying cancer at the time of the appearance of neurological symptoms, he was diagnosed with LEMS as an immune-related adverse event (irAE) induced by atezolizumab. After discontinuing atezolizumab treatment and initiating combination therapy with steroid pulse plus intravenous immunoglobulin, his neurological symptoms improved. Although 18 months have passed since the discontinuation of atezolizumab treatment, there has been neither recurrence of neurological symptoms nor a progression of his cancer without salvage chemotherapy. This is a rare case of LEMS as a neurological irAE induced by atezolizumab. Clinicians must be aware of the potential for LEMS to develop in SCLC patients taking atezolizumab treatment.

## Introduction

Lambert-Eaton myasthenic syndrome (LEMS) is a neuromuscular autoimmune disorder characterized by proximal muscle weakness, reduced or absent tendon reflexes, and autonomic dysfunction [1]. LEMS is also a representative paraneoplastic neurological syndrome (PNS). 47-62% of LEMS patients have associated cancer, of which small-cell lung cancer (SCLC) is the most common [2]. LEMS is thought to be caused by antibodies directed against voltage-gated calcium channels (VGCCs) present on the presynaptic nerve terminal, which reduce acetylcholine release from the presynaptic membrane [3]. Of cases of LEMS comorbid with cancer, only 5% are found to have cancer at the time of LEMS diagnosis, and 86% are found to have cancer after LEMS diagnosis [4]. The diagnosis of LEMS is confirmed by demonstrating characteristic abnormalities in electromyographic studies [1]. Anti-P/Q-type VGCC antibodies are found in 85-90% of LEMS patients [5].

Immune checkpoint inhibitors (ICIs) have become a breakthrough cancer treatment. Notably, the addition of anti-programmed cell death-ligand 1 (PD-L1) antibodies, such as atezolizumab or durvalumab, to chemotherapy in the first-line treatment of extensive-stage small-cell lung cancer (ES-SCLC) has resulted in significantly longer overall survival (OS) than with chemotherapy alone [6,7]. ICIs are also known to cause various immune-related adverse events (irAEs), including neuromuscular disorders. Myositis, Guillain-Barré syndrome and other peripheral neuropathies, myasthenic syndrome, and encephalitis have been reported as the main neurological irAEs [8]. However, LEMS as a neurological irAE is rare. A case of LEMS as an irAE induced by atezolizumab treatment for SCLC is reported.

## Case presentation

**Figure 1:**
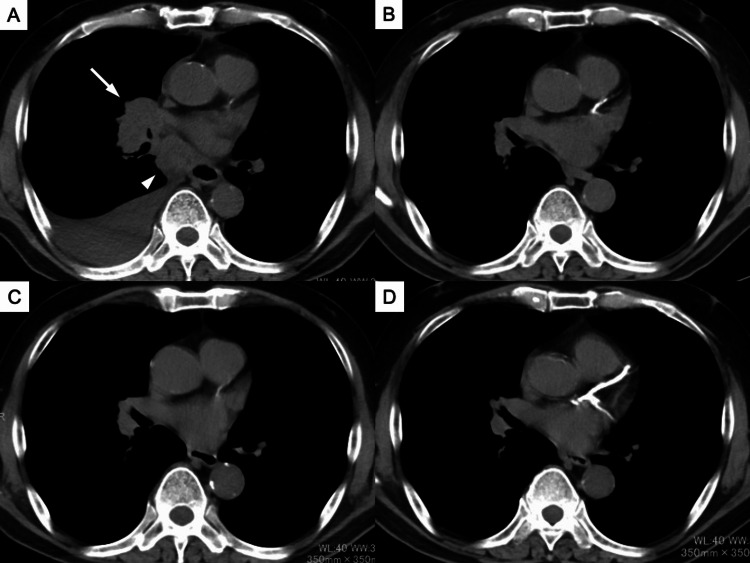
Chest CT shows the primary tumor in the right middle lobe (white arrow), mediastinal lymphadenopathy (white arrowhead), and right pleural effusion at the time of lung cancer diagnosis (A). The primary tumor and mediastinal lymphadenopathy have shrunk, and the right pleural effusion has disappeared after four cycles of combination therapy with atezolizumab plus carboplatin and etoposide (B). Each lesion remains decreased in size or has disappeared during seven cycles of maintenance atezolizumab therapy (C), and even 18 months after the discontinuation of atezolizumab treatment (D).

A 70-year-old man was diagnosed with ES-SCLC with malignant pleuritis (c-stage ⅣA, cT2bN3M1a in the 8th TNM classification) in May 2020. He received four cycles of combination therapy with atezolizumab (dose of 1200 mg) plus carboplatin (area under the curve: 5 mg/mL/min) and etoposide (100 mg/m2 body surface area), which resulted in a partial response (PR) (Figure 1A, 1B) and decreased serum neuron-specific enolase (NSE) and pro-gastrin-releasing peptide (Pro-GRP) levels to the normal range. Subsequently, he received maintenance atezolizumab therapy, and on chest computed tomography (CT), the tumor maintained partial response (PR) during four cycles of maintenance atezolizumab therapy. He experienced no adverse events during this treatment period.

After the fifth cycle of maintenance atezolizumab therapy (8 months after the diagnosis of SCLC and 7 months after starting atezolizumab), he felt muscle weakness in the lower limbs and fatigue, which gradually deteriorated. At the start of the eighth cycle of maintenance atezolizumab therapy (9 months after starting atezolizumab), hypopituitarism (grade 2 Common Terminology Criteria for Adverse Events [CTCAE] v5) and thyroid dysfunction (grade 2 CTCAE v5) appeared as irAEs. It was decided to discontinue maintenance atezolizumab therapy, and he was started on oral hydrocortisone (15 mg/day) and levothyroxine (6.25 μg/day). On a follow-up visit 3 weeks after starting hydrocortisone and levothyroxine supplementation, his blood cortisol levels and thyroid function test were within the normal range. However, he complained of dry mouth, difficulty walking without a cane, and difficulty completing activities of daily living. He was admitted to the neurology department to diagnose and treat neurological symptoms.

On the admission physical examination, his height and weight were 160.5 cm and 58.4 kg, respectively. His temperature, blood pressure, heart rate, and oxygen saturation were 36.6 °C, 116/66 mmHg, 74 beats/min, and 99% (on room air), respectively. Laboratory analyses showed that blood cell counts, biochemical profiles, and tumor marker (NSE and Pro-GRP) levels were within the normal ranges. The tests for antinuclear antibody and anti-SS-A/Ro antibody were negative. On chest CT, the tumor maintained PR even after the seventh cycle of maintenance atezolizumab therapy (Figure 1C).

**Figure 2:**
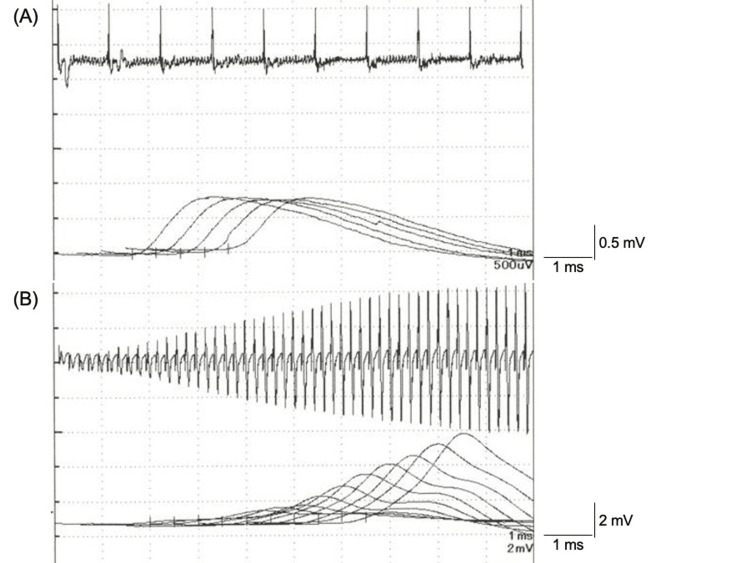
The repetitive nerve stimulation test in the trapezius muscle shows the waning phenomenon with 3-Hz repetitive stimulations of the left accessory nerve. The 9th amplitude of the compound muscle action potential (CMAP) is decreased by 11.9% (0.823 mV to 0.725 mV) compared to that of the first CMAP (A). The repetitive nerve stimulation test in the abductor digiti minimi muscle shows the waxing phenomenon with 50-Hz repetitive stimulations of the left ulnar nerve. The 50th amplitude of the CMAP is increased by 688.5% (0.61 mV to 4.81 mV) compared to that of the first CMAP (B).

On motor examination, bilateral ptosis and muscle weakness that was dominant in the proximal muscles of the lower limbs were noted. Reactivities of the tendon reflexes were reduced bilaterally. He had a slightly waddling gait, dysarthria, and diplopia. He did not have any sensory disturbances. Brain magnetic resonance imaging (MRI) showed no signs of a mass effect, recent infarction, or pathological intracranial enhancement. Anti-P/Q-type VGCC antibody was positive, whereas serum acetylcholine receptor binding antibody was negative. Cerebrospinal fluid examination showed no significant findings. In the nerve conduction study, low-amplitude compound muscle action potentials (CMAPs) were seen at rest in his left accessory nerve and ulnar nerve. The repetitive nerve stimulation test showed the waning phenomenon on 3-Hz repetitive stimulations of his left accessory nerve (Figure 2A) and the waxing phenomenon on 50-Hz repetitive stimulations of his left ulnar nerve (Figure 2B). LEMS was diagnosed as a result of these findings.

The patient was initially started on an oral cholinesterase inhibitor, pyridostigmine (180 mg/day), for 5 days (1 month after discontinuing atezolizumab). However, his neurological symptoms did not improve. Therefore, he was switched to combination therapy with steroid pulse (methylprednisolone 1 g/day for 3 days) plus intravenous immunoglobulin (IVIg) (25 g/day for 5 days). His neurological symptoms improved gradually, and he could walk independently indoors without a cane 2 weeks after starting combination therapy. He was switched to oral prednisolone (30 mg/day) after steroid pulse therapy and discharged home 3 weeks after starting combination therapy. Oral prednisolone was gradually tapered and changed to oral hydrocortisone (15 mg/day) 15 months after starting combination therapy. Although 18 months have passed since the discontinuation of maintenance atezolizumab therapy, there has been neither recurrence of neurological symptoms nor progression of the tumor without salvage chemotherapy (Figure 1D).

## Discussion

A case of LEMS as an irAE induced by atezolizumab treatment for SCLC in which combination therapy with steroid pulse plus IVIg presented a therapeutic effect. Although 18 months have passed since the discontinuation of atezolizumab treatment, there has been neither recurrence of neurological symptoms nor a progression of his cancer without salvage chemotherapy.

Without a validated diagnostic test, it is difficult to determine whether LEMS in SCLC patients on atezolizumab treatment is caused by a PNS of SCLC or an irAE induced by atezolizumab. In the present case, determining the etiology was necessary because atezolizumab treatment could be continued if the LEMS were a PNS of SCLC. In contrast, atezolizumab treatment would need to be discontinued if the LEMS were an irAE induced by atezolizumab. In addition, immunotherapy, including steroid and immunosuppressant therapies, could facilitate tumor progression in a LEMS patient with a PNS of SCLC [9]. In consultation with a neurologist, the focus was on the onset time of LEMS and the state of his underlying cancer at the time of the appearance of the neurological symptoms. According to a previous study, SCLC was found before the diagnosis of LEMS in only 7% of LEMS patients with SCLC, and SCLC was detected within 1 year after the diagnosis of LEMS in 96% of these patients [10]. Neurological irAEs occur after a median of 5.5 cycles of ICIs with a range of 1-20 cycles [11]. In the present case, his neurological symptoms appeared after completing 9 cycles of atezolizumab (8 months after the diagnosis of SCLC). Although it is known that the remission of SCLC usually results in the improvement of neurological symptoms in paraneoplastic LEMS [12], in the present case, cancer had stabilized in PR at the time of the appearance of neurological symptoms. Based on these facts, he was diagnosed with LEMS as an irAE induced by atezolizumab. Furthermore, no progression of his cancer without salvage chemotherapy, even after discontinuing atezolizumab treatment, might favor atezolizumab as the cause of LEMS. However, whether atezolizumab treatment caused the LEMS or uncovered pre-existing paraneoplastic LEMS is still unclear.

The present case showed the therapeutic effect of combination therapy with steroid pulse plus IVIg for neurological symptoms due to LEMS as an irAE induced by atezolizumab. The general principle of treatment for LEMS caused by a PNS of SCLC is treating SCLC with chemotherapy, radiotherapy, or surgery. 3,4-aminopyridine (DAP) or cholinesterase inhibitors are used to improve clinical symptoms. If 3,4-DAP or cholinesterase inhibitors are ineffective, steroid and immunosuppressant therapies can be used. IVIg or plasmapheresis is recommended when steroid and immunosuppressant therapies have an insufficient effect. Rituximab is considered for refractory LEMS patients [9].

On the other hand, effective treatment for ICI-induced LEMS remains to be established. In general, the treatment for ICI-induced neurological irAEs depends on the severity of the symptoms. For all mild neurological symptoms attributed to ICIs, ICI therapy should be discontinued, and steroid therapy should be instituted. IVIg or plasmapheresis should be considered in cases of lack of response to steroid therapy [13]. To the best of our knowledge, there have been seven case reports of ICI-induced LEMS, including four cases of SCLC [14-17], one case of a neuroendocrine tumor with small cell features [18], one case of squamous cell carcinoma [19], and one case of squamous cell carcinoma and large cell neuroendocrine carcinoma [20]. All of them were patients with lung cancer, and the causative agents were atezolizumab, pembrolizumab, and nivolumab in two cases each and nivolumab plus ipilimumab in one case. Although various treatments, including 3,4-DAP, pyridostigmine, steroid, azathioprine, IVIg, or rituximab, were administered in these seven cases of ICI-induced LEMS, treatment outcomes were inconsistent. In particular, atezolizumab was used in two of four cases of ICI-induced LEMS in SCLC patients. Krishnan et al. showed that IVIg, prednisolone, and amifampridine were ineffective for neurological symptoms [15]. Kunii et al. showed that steroid pulse therapy was ineffective, but IVIg with steroid maintenance was effective for neurological symptoms [17]. In the present case, neurological symptoms improved after initiating combination therapy with steroid pulse plus IVIg. Further studies are needed to understand the optimal management of ICI-induced LEMS.

## Conclusions

A case of LEMS as an irAE induced by atezolizumab treatment for SCLC was described. Anti-PD-L1 antibodies combined with chemotherapy have become the first-line treatment of ES-SCLC, which may increase the number of cases of ICI-induced LEMS. Since the tumor type is almost always SCLC in patients with LEMS, considering the onset time of LEMS and the state of underlying cancer at the time of the appearance of neurological symptoms may help distinguish ICI-induced LEMS from paraneoplastic LEMS. Although effective treatment for ICI-induced LEMS remains to be established, combination therapy with steroid pulse plus IVIg may be one of the options for LEMS induced by atezolizumab treatment for SCLC. Clinicians must be aware of the potential for LEMS to develop in SCLC patients on atezolizumab treatment.
